# Study of energetic properties of different tree organs in six *Olea europaea* L. cultivars

**DOI:** 10.1038/s41598-021-96436-y

**Published:** 2021-08-23

**Authors:** G. Sala, T. Caruso, F. P. Marra, F. Zafonte, A. Amico Roxas, B. Schiavo, A. Galia, A. Brunori, F. Dini, L. Regni, P. Proietti, T. La Mantia

**Affiliations:** 1 Department of Agricultural, Food and Forestry Science, University of Palermo, Viale delle Scienze, Ed. 4, 90128 Palermo, Italy; 2 Department of Landscape Design and Sustainable Ecosystems, Peoples’ Friendship University of Russia, Miklukho-Maklaya str., 6, Moscow, Russia; 3 Department of Plant Sciences, University of California, Davis, One Shields Ave., Davis, CA 95616 USA; 4 Department of Engineering, Chemical, Biochemical and Materials Engineering, Hydraulics, University of Palermo, Viale delle Scienze, Ed. 6, 90128 Palermo, Italy; 5 Department of Agricultural, Food and Environmental Sciences, University of Perugia, Borgo XX giugno, 74, 06121 Perugia, Italy; 6 Department of Architecture, University of Palermo, Viale delle Scienze, Ed. 8, 90128 Palermo, Italy

**Keywords:** Environmental impact, Agroecology

## Abstract

Pruning is an important horticultural practice for the management of olive orchards (*Olea europaea* L.) that generates a considerable amount of residues every year. Olive orchards are increasingly expanding beyond the Mediterranean Basin to new growing Countries (Australia, California, Argentina, Chile and Uruguay) and this will certainly lead to larger availability of pruning material. Currently, the interest in use of olive tree pruning residues for energy purposes is increasing but unfortunately, the information on the differences among organs of the tree, in terms of calorific value and ash content, is scarce. Another unknown aspect is the effect of cultivar vigour on dry matter partition among different tree organs, these are important traits to establish the energetic quality of pruning residues. The aim of this research was to study energetic aspects of six olive cultivars, largely grown in the Sicilian olive industry and characterized by different vigour. The trees taken into consideration in the study were selected in an experimental orchard to avoid any effect due to differences in environmental conditions and management. The energetic characteristics, calorific value and ash content, were evaluated for the various tree organs particularly shoots, leaves and branches; also root system was evaluated, although the roots can only be used once the trees are uprooted. Significant differences were observed in the calorific values among the different tree organs and the cultivars. Regarding the ash, shoots and leaves showed the highest content with respect to the other organs, thus causing a possible tendency in slagging with fouling and corrosion of boiler components.

## Introduction

One of the challenges for the coming future is to increase renewable energy to 32% by 2030^1^. Biomass is one of the most important sources of renewable energy and vegetal wastes have a crucial potential to produce sustainable energy from renewable fuels^2,3^. The rapid development of the bioenergy sector has produced a growing demand for biomass among which pruning residues represent a huge and low cost resource; moreover, the use of biomass for the production of energy can significantly contribute to job creation and economic development of rural areas’ economies^3,4^.

In the Mediterranean Basin, a large amount of agriculture by-products are extensively produced every year^2^. Italy is characterized by an extensive fruit orchard surface (2,140,145 ha)^5^ and the estimated crops residues is around 1,483,905 t/y^3^. The residues of agricultural industry are one of the major sources of biomass, and studies aimed to understand the energetic characteristics of biomass, in particular pellet, are considerably increasing in recent years^6–8^. Many researchers have evaluated the heating value of different wood species^8^ but only few are the studies on fruit trees species that focus on the energy content of the various tree organs as affected by the genetic traits^9,10^. Plant organs (leaves, branches and roots) are characterized by different morphological functional traits, furthermore trees adopt different strategies to distribute carbohydrates, thus energy, in the different organs^9^.

In the last two decades, olive industry (*Olea europaea* L.) has spread worldwide (10.5 million ha^11^) also for the well-known health benefits of olive oil in the diet^12^. Olive tree is one of the best adapted crop in marginal sub-humid and semi-arid zones of the Mediterranean area. Italy is the second worldwide producer after Spain^11^ (2,320,392 ha of olive orchards ISTAT, 2020) and Sicily is the third regions in Italy for surface (166,006 ha^5^), after Apulia and Calabria. Among olive orchard management practices, pruning aims to remove unproductive and/or exhaust organs, to adjust crop load to the photosynthetic potentiality of the vegetative organs, to stimulates new vegetative growth and enhances air circulation within the canopy and light interception by the inner foliage^13^. Usually, in traditional olive orchards, pruning is carried out every two years, mostly after the on year (heavy crop load) in order to remove the surplus branches, and to eliminate the exhaust part of the branches, that are abundant in the inner and lower part of the canopy. The above mentioned horticultural practice produces a large quantity of pruning by-products^13^. In the last 30 years, the percentage of new orchards has increased by 30% in the world^14^ and there is a tendency to replace old olive orchards and to establish new ones with high density plantation^15^. To increase orchard productivity and allow the mechanization of the harvest, the new planting systems are characterized by higher tree density, rectangular design and hedgerow training system^16^. In order to maintain efficient the new systems, it is required annual and severe pruning that in turn generates a huge amount of residues, which must be disposed of^15^. Due to the long life of the olive tree, a large part of the old olive orchards are based on trees older than 100 years, characterized by low efficiency in olive production and high pruning residue per tree. A large part of the tree in the traditional orchard is usually represented by trunks and large woody branches rather than fruiting organs (e.g. current year/one-year-old shoots and leaves). Due to the low orchard productivity and the high demand for hand labor (mainly for pruning and harvesting), the primary sector of olive oil chain needs to be renewed to increase their economic sustainability. The conversion of traditional olive orchards into new systems (i.e. high and super high density system) produced huge amount of olive wood and pruning residues per unit area and currently in Italy, the annual amount of olive pruning residues has been estimated to 2.85 Mt^17^.

The pruning residue from the olive tree is a key factor of the olive grove system, that could also be taken into consideration and evaluated^18^; this material is additional biomass to use as a source of energy and play a role in supplying bioenergy plants with renewable fuel, especially in rural areas. Usually, in the Mediterranean area, the management of pruning residues is more a disposal problem, rather than an opportunity for additional revenue. Indeed, the residues are generally burned in the orchard causing both economic and environmental problems. Traditional uncontrolled combustion of olive pruning in the field caused environmental problems as contaminants emissions, risk of fires and loss of a source of energy^19^. Also, this material is ground and the resulting chips scattered over the field with a high cost for the farmers. This practice increases the fertility of the soil with rise of soil organic carbon but only the thin components cause this effect^20^. What is needed to maximize environmental benefits is an integrated and organized waste management system as required by the European legislation^21^. It should also be considered that Italian legislation has revised the legislative “status” of pruning residues which today are no longer considered waste (Italian Law 37/2019).

Usually, the olive pruning material includes different organs: wood, leaves and shoots; the weight fraction of these different organs ranges 25–45% for the leaves, 25–38% for the wood and 16–50% for the shoot^22,23^. These percentages may largely vary depending on irrigation management (rainfed or irrigated orchards), tree age, vigour and productivity and local pruning practice. An important factor that define the biomass is the tree vigour which represent the annual increase of the vegetative parts of the tree^13^.

To estimate the energy content, the calorific value (or heating values) is used as a measurement of enthalpy released during combustion, with an excess of oxygen at fixed pressure and temperature and it can be considered an important property of pruning residues, which reflects the ability of the tree species to store the solar energy in the biomass during photosynthesis^24^. The ash is the total amount of mineral content of a tree, which is determined by burning a given quantity of the tree. The ash content is an important parameter affecting the performance of the combustion because a high inorganic fraction increases the problem of fouling (forming deposits on combustor surfaces) and slagging^24^. A high ash content reduces also the heating value of the pruning residues and negatively affects milling and pelleting equipment^25^.

Although in recent years the studies on energy production from residual biomasses are increasing, usually they do not consider the calorific value of different organs of the tree. Biomass is an available removable energy source, characterized by high energetic potential; the calorific value of different organs of plant and cultivar is critical for biomass utilization as background for new orchards and for utilization of waste of orchards already present. Investigate variation within trees is important in order to provide recommendations for the most efficient use of the trees’ organs.

The aim of this research was to study the combustion characteristics (calorific value and ash contents) of the various organs of olive tree belonging to six cultivars. These cultivars were chosen with different tree vigour and canopy architecture (related to the partitioning of different organs within the tree).

## Materials and methods

### Experimental site

The field activity was carried out during 2015; samples of pruning residues were collected from the winter pruning material of thirty-year-old olive trees (*Olea europaea* L.) grown in an experimental orchard located in the Southern coastline of Sicily (Castelvetrano, 37° 35′ 13″ N, 12° 53′ 47″ E; 50 m a.s.l.). Trees were trained into a globe-vase shape and spaced 7 m × 5 m apart (286 trees ha^−1^) under deficit irrigation regime (around 500 m^3^/ha/year) and pruned every year. The climate at the experimental site is typically Mediterranean and it is characterized by hot and dry summers and mild winters with an annual average rainfall of 500 mm^26^. The soil is sandy clay loam with 7.8 pH. The experimental orchard consists of an ex-situ collection of Sicilian olive trees cultivars, plus some other cultivar assumed as national references^26^.

### Plant material

Samples were collected from six different cultivars. Five of those were Sicilian cultivars: Calatina, Biancolilla (both characterized by low vigour)^27^, Verdello (medium vigour), Cerasuola and Olivo di Mandanici (OdM throughout the text) (high vigour^26,28^ and unpublished data). The sixth one, Leccino, with high vigour, is one of the most common cultivars grown in Italy. Three trees for each cultivar were selected for the study; samples were collected from different organs of the trees namely: twigs (shoots and branches < 5 cm diameter) and foliage; branches (> 5 cm diameter) and, after the trees were uprooted, root system. Roots were collected only from four cultivars (OdM, Leccino, Cerasuola and Verdello) because the techniques to extract the root was expensive and time consuming; furthermore the collection of this organ was interrupted because after the preliminary tests no differences were found among the cultivars. All samples were collected from the canopy after fruit harvest and during pruning period (February); roots samples were collected from uprooted trees by excavation with a bulldozer all sides around each tree, in order to collect mostly of the root system.

#### Statement

All material collection of plant material complied with relevant institutional, national, and international guidelines and legislation.

### Samples preparation and determination of energetic properties

Three trees of each cultivar were cut down and separated into three component (twig and foliage, branches and root). The laboratory activity was carried out during 2020. Each sample representing an organ of tree were dried in an oven at 105 ± 2 °C until a constant weight was reached. The samples were grounded using a laboratory hammer mill (Retsch ZM1) in order to reduce the dry matter smaller than 1 mm of size, samples were then manually homogenized for each organ to obtain a homogeneous sample.

The calorific value of the sample was determined using an adiabatic calorimeter. Gross calorific values (or higher heating value) were measured following CEN/TS 14918: 2005, Solid Biofuels—Method for the Determination of Calorific Value by using a Parr 6200 Isoperibol Calorimeter. The ash content was determined according to the UNI EN ISO 14775 Solid Biofuels—Determination Of Ash Content 2010^29^. Samples were burnt in a muffle furnace at 550 °C and each measurement was repeated three times to estimated experimental uncertainty. The residual ash was weighed in an electronic weighing scale (precision +/- 0.1 mg), and ash percentage was determined on a dry basis (wt % on dry basis).

### Statistical analysis

For the analysis of heat values a linear mixed effect model was used with cultivar as fixed factors and trees as a random factor. Tukey’s HSD test was performed to separate means when ANOVA results were significant (*P* < 0.05). All data were analyzed using the R statistical software^30^.

## Results

The calorific value ranges from 20.83 to 18.41 MJ/kg in twig and leaves fractions, from 20.31 to 19.27 MJ/kg in branches and from 19.20 to 19.88 MJ/kg in the root system (Table 1). Significant differences (*P* < 0.05) in twig and foliage fraction resulted among cultivars. The calorific value in Calatina was significantly higher (20.83 MJ/kg) with respect to Verdello (18.41 MJ/kg). No significant differences were found between Biancolilla and Leccino. The high vigour cultivars, OdM and Cerasuola, showed the same calorific value. Significant differences were also found among cultivars for calorific value of branches with Calatina evidencing the highest value (20.31 MJ/kg), while Verdello (19.27 MJ/kg) the lowest. No differences were found among the other cultivars (Biancolilla, Cerasuola, OdM and Leccino). Finally, no differences were observed in the calorific values of the root system (average value of 19.44 MJ/kg).

**Table 1:** Mean calorific values (MJ/kg) of dry matter measured in the different organs’ in the cultivars studied. Means within columns with the same small letters are not significantly at *P* < 0.05 (n = 3), SD is standard deviation. For the root system there are not small letters because few samples.

Cultivars	Vigour	Twig and foliage		Branches		Root system	
		Mean	SD	Mean	SD	Mean	SD
Calatina	Low	20.83^a^	0.16	20.31^a^	0.84		
Biancolilla	Low	20.22^ab^	0.09	20.09^ab^	0.31		
Verdello	Medium	18.41^c^	0.17	19.27^b^	0.59	19.88	0.67
Cerasuola	High	19.98^b^	1.30	19.92^ab^	0.94	19.45	0.68
OdM	High	19.79^b^	0.45	19.35^ab^	0.19	19.25	0.15
Leccino	High	20.10^ab^	0.45	19.64^ab^	0.87	19.20	0.49

Although twig and foliage fraction showed the highest ash content (5.7%) followed by root (2.4%) and by branches (1.1%), no differences among the cultivars were observed in the different organs of the trees (Table 2).

**Table 2:** Ash (%) on dry basis. Means within columns with the same letter are not significantly at *P* < 0.05.

Cultivars	Twig and foliage	Branches	Root system
Calatina	6.27^a^	1.58^a^	–
Biancolilla	5.53^a^	0.96^a^	–
Verdello	4.79^a^	0.98^a^	1.76^a^
Cerasuola	5.57^a^	0.99^a^	2.99^a^
OdM	5.72^a^	0.71^a^	2.78^a^
Leccino	5.88^a^	1.33^a^	2.53^a^
*Mean*	5.67	1.12	2.37

Finally, the average weight of pruning material dry matter available each year per hectare was about 3 t ha^−1^ and 5 t ha^−1^ for the low and high vigour cultivars respectively.

## Discussion

Our results suggest that the calorific values of different tree organs are ranked as following twig and foliage fraction > branches > root system. Previous studies on the calorific value of olive pruning residues did not analyse the effect of different organs and/or different cultivar. Sirna^31^ reported as calorific value for olive pruning material 19.4 MJ/kg; Requejo et al.^32^ reported in their study heating values of two pruning fractions: wood with 19.1 MJ/kg, and leaves and twigs (< 1 cm in diameter) less than 18.69 MJ/kg. For thin branches (< 5 cm diameter) and leaves of Picual cultivar, Cuevas et al.^33^ measured values of 17.4 MJ/kg. Another study on pellet obtained from olive pruning residuals reported values of 19.64 MJ/kg for the leaves and of 17.53 MJ/kg for wood^34^. All these values are lower than the ones we observed in this study.

The higher value reported for leaves is confirmed also in our study where the calorific value of twigs and leaves was higher than 2% with respect to the wood. The calorific value of foliage is generally the highest among organs of the plant because leaves are the most active organs and contain many high-energy compounds. The olive leaves contain a very high total extractives content (ranging to 35–40%)^35,36^, they include organic compounds (as waxes, alkaloids, simple sugar, phenolics, proteins, sugar, gums, resins, terpenes, starches, glycosides, saponins and tall oil) with function in tree metabolism, defence mechanism and energy reserves^37^, and they showed the highest calorific value compared to the lignocellulosic composition. Also the percentage of lignin was found higher in olive leaves than in the other organs^36^ and lignin has considerably high combustion heat^38^.

The difference in calorific values in olive trees between above- and belowground organs, suggests that roots had lower calorific value; previous studies on other species^9,10^ have shown that differences among several fractions are higher in aboveground organs (leaf, branch, and trunk) than in underground organs (root). The calorific value of the roots is generally lower with respect to organs photosynthetically active as leaves and shoots. Roots, besides being devoted to absorbing water and nutrients from the soil have to guarantee energy to sustain the growth of the other organs of the plant and for this function their content in starch, fats, crude proteins and other substances is higher than in other organs of the trees^10^ so the calorific values are relatively lower. Moreover the calorific value is connected to the lignin content^39^ and likely in the root the lignin content is low; further analysis is necessary.

Regarding the effect of the cultivar, the highest calorific value obtained from cultivar Calatina (characterized by low vigour) for all the organs of tree seems to show the effect of vigour. These differences might depend on the different components for example lignin^39^, extractives and oil content of the leaves. Also the different growth rates of the cultivar might have an effect on the composition of organs and therefore the calorific value. The cultivar Verdello with medium vigour showed the lowest calorific value.

With regard to the ash, there is substantial diversity in the content of ash among the different fractions of the tree (Table 2). The ash data appears fundamental in selecting the best biomass for energy conversion because a high ash content negatively affects the calorific value and causes the formation of vitrified surfaces in the furnace that reduces the efficiency of the heat exchange systems and the durability of the equipment^25^. The ash content of olive fraction in this study ranked in descending order twig and foliage fraction > root system > branches. Twig and leaves showed the highest ash content, and similar values were obtained by other studies^36,40^ who found that the content of ash was 5.5% for olive tree pruning residues. Compared to the limit values for class “broad-leaf virgin wood materials, logging residues” of UNI EN ISO 17225-1 (2014), the ash content for branches and root system of all cultivars are in compliance with the Technical Standard since the ash content is less than 5% on a dry basis, that represent the maximum references acceptable value; twig and leaves were slightly higher but still close to the typical variation.

The quantification of pruning is a process long and expensive; the estimation of the quantity of pruning by-products depends on many factors: tree age and size, orchard management, particularly planting density (e.g. trees space), pruning intervals (annual/biannual) and severity criteria (training, sanitary, rejuvenation pruning), cultivars and irrigation supply. The production of biomass for annual pruning of trees older than 40 years is largely higher respect to younger tree (respectively 2.3 t ha^−1^ and 1.4 t ha^−1^)^41^. Spinelli and Picchi^42^ quantified the pruning residues from 4 to 11 over dry t ha^−1^. This data of annual pruning residue was confirmed also in the orchards chosen in same area of this study during the LIFE15 OLIVE4CLIMATE project, where the pruning weight was 2.9 dry t ha^−1^ (in coastal orchards), whereas pruning weight was larger than 3.05 of dry t ha^−1^ in inland orchards (https://olive4climate.eu/); this value is confirmed in other orchards where an average of 3 t ha^−1^ of olive-pruning is generated^43^.

The data that estimate the energy obtainable from the pruning residues of the olive groves in Sicily are outdated, Beccali et al.^14^ for example considered the potential biomass from vineyard and olive crops in 1000 kt/year and they assessed that only a portion between 25 and 50% could be collect in the coming years depending on the development of facilities for the use of biomass. Today, due to the changes that have occurred in Sicilian olive orchards the quantity of energy that can be obtained is much higher.

Assuming a potential pruning residues production of 3 t/ha yearly and the presence of about 166,000 ha of olive tree groves in Sicily, a total amount of 483,000 t of pruning residues per year could be potentially available as renewable energy source, with a corresponding energy value of about 9.6 × 10^9^ MJ each year. As a comparison, this energy amount represents tenfold the energy derived by diesel oil used in Sicily for domestic heating in the year 2014^44^.

## Conclusion

The evaluation of the calorific value and ash of different cultivar and different fractions of olive tree was carried out to investigate the potential use of olive pruning residues for energy generation. From the utilization point of view the leaves and twigs of Calatina cultivar have a higher heating value in comparison with other cultivars; but the presence of leaves increases the amount of ash content. Twig and foliage fraction showed the highest ash content, thus, causing a tendency to slag, foul and corrode equipment boiler components during the combustion process. In all cultivars, branches were more suitable than leaves and roots in term of low ash concentration. This problem can be solved by collecting the pruning residues from the ground after the leaves have fallen or with the use of defoliation machines; this would reduce the ash content and allow a return to the soil of organic matter.

The exploitation of olive residual biomass for energetic purposes could represent an integrative income for the farmer. Indeed, olive pruning residues with other orchard residues could represent an important biomass resource for energy production but in the supply chain the key is to identify the best technologies to harvest pruning residues.
